# A 1RM Strengthening and Exercise Programme for the Treatment of Knee Osteoarthritis: A Quality-Improvement Study

**DOI:** 10.3390/jcm12093156

**Published:** 2023-04-27

**Authors:** James Creasey, Jo Masterman, Gregory Turpin, Richard Stanley, Tikki Immins, Louise Burgess, Thomas W. Wainwright

**Affiliations:** 1 Physiotherapy Department, University Hospitals Dorset NHS Foundation Trust, Bournemouth BH7 7DW, UK; 2 Orthopaedic Research Institute, Bournemouth University, Poole BH12 5BB, UK; 3 Clinical & Rehabilitation Services Department, AECC University College, Bournemouth BH5 2DF, UK

**Keywords:** knee, osteoarthritis, exercise, strengthening, physiotherapy

## Abstract

**Background:** The Kneefit programme is a 12-week strengthening and exercise programme, personalised using body-weight ratios, for people with knee osteoarthritis. **Objectives and Design:** This quality-improvement study was conducted to evaluate the effectiveness of the programme for managing symptomatic knee osteoarthritis. **Methods:** The Kneefit programme was delivered between 20 August 2013 and 7 January 2014 and included six weeks of supervised strengthening, balance, and cardiovascular exercise in a group at the local hospital, followed by six weeks of unsupervised exercise. Leg-press and knee-extension 1RM scores were assessed at baseline, six weeks, and twelve weeks. In addition, patient-reported outcome measures (Oxford Knee Score, EQ5D, Patient Specific Function Score (PSFS)) were assessed. Wilcoxon Signed Rank tests were used to evaluate the changes from week 1 to week 6 and week 12. **Results:** Thirty-six patients were included at baseline and at six weeks, and 31 patients completed their twelve-week assessment. Statistically significant improvements were found at 6 and 12 weeks for change for the Oxford Knee Score (median change: 4.0, IQR 4.0 to 9.0, *p* < 0.001 and 4.0, IQR 0 to 8.0, *p* < 0.001), EQ5D-5L (median change: 0.078, IQR 0.03 to 0.20, *p* < 0.001 and 0.071, IQR 0.02 to 0.25, *p* < 0.001) and the PSFS (median change: 1.3 IQR 0 to 2.6, *p* = 0.005 and 2.3 IQR −0.3 to 3.3, *p* = 0.016). In addition, significant improvements were found for 1RM leg-press and knee-extension scores on both the affected and unaffected legs. **Conclusion:** The Kneefit programme was successful at improving both functional and strength-related outcome measures in patients with knee osteoarthritis. Our findings suggest that tailoring strength exercises based on the 1RM strength-training principles is feasible in this population.

## 1. Introduction

Osteoarthritis is a chronic degenerative joint disorder that typically presents as joint pain accompanied by varying degrees of functional limitation and reduced quality of life. It is estimated that 18.2% of people aged 45 years and over in England have osteoarthritis of the knee, equating to around 4.5 million people [1]. Globally, projections based on demographic trends suggest that the prevalence of osteoarthritis will continue to increase in line with the world’s ageing population and the global obesity epidemic [2]. Locally, in Dorset (England), there is a high percentage of adults aged 65 or older (28.6%), almost a two-fold increase on the national average for England and Wales (18.3%) [3]. The local hospital performed 1521 total knee replacements between 1 April 2018 and 31 March 2021, over three times the national average of 554 knee replacements in the same time period [4].

As there is no known cure for osteoarthritis, non-surgical management for people with symptoms not yet severe enough for surgery focuses on alleviating pain and maximizing function by addressing modifiable aspects of the condition. The National Institute for Health and Care excellence (NICE) guidelines recommend three core treatments to manage osteoarthritis: education and advice; exercise; and weight loss where necessary [5]. These guidelines assimilate with recommendations from the American Academy of Orthopaedic Surgeons (AAOS), who recommend that patients with symptomatic knee osteoarthritis participate in self-management programmes, strengthening, low-impact aerobic exercise, neuromuscular activation and weight loss where necessary [6]. Similarly, the Osteoarthritis Research Society International (OARSI) have deemed structured, land-based exercise programmes, dietary weight management in combination with exercise, and mind–body exercise (such as tai chi and yoga) to be core treatments for knee osteoarthritis [7].

Systematic review evidence has highlighted the benefits of aerobic exercise, strength training, neuromuscular exercise, and mind–body exercise such as tai chi and yoga [8]. However, while therapeutic exercise is universally recommended as a first-line treatment for patients with knee osteoarthritis, there is little specific guidance for clinicians on its implementation; therefore, there is a need to develop local models of care to deliver the core treatments recommended by the NICE, OARSI and the AAOS. For example, there is no specific guidance on the type of exercise, dose, intensity, progression or delivery method [9], with systematic reviews identifying exercise as an effective treatment regardless of the methods of delivery (i.e., individual, group or home-based) [10], or the types of exercise undertaken [11].

Effective models of care are required locally to deliver the non-surgical interventions for knee osteoarthritis. In Bournemouth, the standard care for patients reporting knee pain to their general practitioner (GP) can be inconsistent, in that they may receive general advice, advice on analgesia and/or physiotherapy, and self-management. Prior to this quality-improvement initiative, standard care within the local hospital involved a one-hour education class and advice session (“The osteoarthritis knee group”) that was considered ineffective at managing osteoarthritis symptoms, particularly for those younger patients seeking advice on exercise doses and how to manage their symptoms while continuing with their careers. Consequently, discussion amongst the local physiotherapy team led to the development of the “KneeFit” group, a supervised exercise and strengthening programme aimed at improving the strength of the muscles around the knee joint. The quality-improvement project described here was conducted to evaluate the effectiveness of the KneeFit programme for managing symptomatic knee osteoarthritis in patients from Bournemouth, Dorset, by evaluating patient-reported outcome measures (PROMs) and lower-limb strength before and after the programme.

## 2. Methods

### 2.1. Context

This is a quality-improvement study, reported in accordance with the Standards for Quality-Improvement Reporting Excellence (SQUIRE) 2.0 guidelines. SQUIRE is intended for reporting the range of methods used to improve healthcare and provides guidance on how to share these discoveries [12]. The Template for Intervention Description and Replication (TIDieR) (Supplementary File S1) and Consensus on Exercise Reporting Template (CERT) (Supplementary File S2) were used to describe the exercise interventions implemented [13,14].

### 2.2. Intervention

The Kneefit programme is a twelve-week exercise programme, delivered for the first six weeks in a supervised group setting at the physiotherapy department of the Royal Bournemouth Hospital, followed by six weeks of unsupervised, progressively graded exercise. Aside from education on exercising, there were no non-exercise components in the programme. The KneeFit programme was developed, reviewed, and guided by the Plan, Do, Study, Act (PDSA) model of continuous service improvement [15] (Table 1), the first version of the programme is reported in this quality-improvement study.

#### 2.2.1. Participants

Patients were referred to the programme from the physiotherapy departments of two local hospitals (The Royal Bournemouth Hospital and Christchurch Hospital) or from the orthopaedic outpatients department of the Royal Bournemouth Hospital between 20 August 2013 and 7 January 2014. Physiotherapists at these sites received training on the purpose and content of the Kneefit programme, its criteria for inclusion, and the referral process. Patients were considered for the programme if they: (i) had a diagnosis of knee osteoarthritis as per NICE guidelines (ii) had levels of pain that did not prevent exercise participation; and (iii) had a minimum of 100 degrees knee flexion. Patients were excluded from recruitment if they had: (i) an uncontrolled cardiovascular or respiratory condition, (ii) uncontrolled diabetes, (iii) inflammatory disease (rheumatoid arthritis, ankylosing spondylitis), (iv) functional limitations that precluded the use of exercise equipment; or (v) high levels of frailty.

#### 2.2.2. Delivery

The supervised exercise programme was delivered by an NHS AFC band 6–7 musculoskeletal physiotherapists with experience of treating knee osteoarthritis, and supported by two exercise leaders (band 4, with an exercise qualification). The delivering physiotherapists received training from senior physiotherapists on the content and structure of the programme. The programme continued in a ‘two-in, two-out’ format maximising the programmes capacity. The class size consisted of 12 patients to optimise the use of equipment and staffing ratios, given the resources available. The class was 60 min long to allow for adequate time to complete the exercise programme and included rest time, and the first session included an additional 45 min for testing and instruction/demonstration of the exercise circuit.

#### 2.2.3. Exercise Prescription

The supervised component of the Kneefit programme involved a circuit-based course incorporating ten exercises aimed at improving strength and cardiovascular fitness as per NICE guidelines for the management of lower-limb osteoarthritis [5]. Exercises were selected to target the major muscle groups of the lower limb and included cycling, rowing, cross-training, leg press, leg extension, hamstring curls, resisted side stepping, bridging, balance exercises, and calf raises. Patients were prescribed strengthening exercises in three sets of between 8–12 repetitions in accordance with the ACSM’s guidance of training for strength [16] but adjusted to the patient’s individual needs as necessary. The level of resistance load was determined by the patient’s 1RM results on initial testing. This was set to 75% of their 1RM as per the guidance set by the ACSM but could be adjusted to suit the patients depending on their ability to complete the desired sets and repetitions or levels of pain related to the resistance load of the exercises [16]. Resistance load and any pain experienced were recorded on paper sheets, and then used to inform intensity progression or regression during subsequent sessions. Patients were encouraged to increase the load if they could complete three sets of 12 repetitions easily and with minimal knee symptoms. Likewise, if patients struggled to complete their prescribed exercise, resistance would be regressed. Rest time between sets was 1 min, the minimal time recommended by the ACSM [16] but the maximal practical rest time for the class format.

Cardiovascular exercise consisted of cycling, rowing and a cross trainer adjusted for the individual, and intensity was prescribed using the Borg Scale (1–10 version, level 7–8, vigorous activity) to encourage a high-intensity workout. In addition, the programme included balance and proprioception training, using wobble boards, BOSU balls, rocker boards and balance tasks, as evidence suggests that exercising on unstable surfaces improves the symptoms of knee osteoarthritis [17]. The full programme can be found in Supplementary File S3.

Once the six weeks of supervised exercise was completed, patients were provided with a graded exercise programme (Supplementary File S4) based on the exercises performed in the class. Participants were given different levels of exercise, and decision rules for progression were included on the exercise sheet. For example, “once you can do 25 repetitions, move onto the next level.” Patients were offered unsupervised use of the hospital gym for six weeks, independent use of their own gym for six weeks, or unsupervised, home-based exercise for six weeks. Patients returned to the hospital for a twelve-week review once the six-week home exercise programme was complete.

### 2.3. Motivation Strategies

Patients were asked to identify up to five important activities that they had difficulty performing because of their knee osteoarthritis during completion of the Patient Specific Function Scale (PSFS). These activities were used as part of a personalised goal-setting process pre-programme, to increase motivation to complete the programme. These goals were re-reviewed during the six- and twelve-week follow ups.

### 2.4. Fidelity

During their first session, participants received training and demonstrations of the exercise circuit. In addition, each group class was supervised by one physiotherapist (band 6–7) and two exercise leaders (band 4, with an exercise qualification), stationed by resistance equipment, to ensure patients were exercising at their prescribed intensity, and performing the exercises correctly. Before starting unsupervised exercise during weeks 6–12, patients received training on how to complete the prescribed exercises, and an exercise sheet containing diagrams of the exercises (Supplementary File S4).

### 2.5. Adherence

Adherence was monitored through the group class by the physiotherapist and exercise leaders. In addition, pain was monitored during exercise, so that intensity could be adjusted accordingly, and to reduce the risk of drop-outs. During weeks 6–12, patients were asked to complete an exercise diary (Supplementary File S5), to encourage and monitor adherence to the home-exercise programme.

### 2.6. Study of the Intervention

Patients completed an assessment at week one, week six, and at week twelve, to assess the effect of the Kneefit programme on their knee strength and symptoms. Assessments were performed by physiotherapists and physiotherapy assistants who were trained to assist in the data collection process. Based on their twelve-week assessment, patients were either: (i) discharged with advice on ongoing self-management of symptoms based on NICE guidance; (ii) discharged with an exercise referral to a local provider; or (iii) returned to their referring therapist to discuss further management options. Patients who failed to attend the twelve-week follow-up were contacted by phone to attempt to obtain outcome measure data and were offered the management options.

### 2.7. Measures

Age, weight, and affected side(s) were recorded for all patients. In addition, patients completed one repetition max (1RM) testing, used to prescribe strengthening exercise, and as a measure of progression, in addition to three patient-reported outcome measures (PROMs) (EQ5D-5L Oxford Knee Score, The Patient-Specific Function Scale). These measures were completed at baseline (week one), week six (following completion of the supervised element of Kneefit) and at week twelve (following completion of the unsupervised exercise).

#### 2.7.1. One Repetition Max (1RM) Testing

Patients performed one repetition max (1RM) testing of knee extensions and leg presses on both their affected and non-affected sides, before and after the programme, as a measure of strength improvement and a method to prescribe strengthening exercises. One-repetition maximum tests were performed on the leg-extension (Technogym Selection Med Leg Extension, Technogym, Bracknell, UK) and leg-press machines (Cybex Eagle Leg press 11040.29, Cybex, Northamptonshire, UK) in the hospital gym. Patients warmed up for 10 min on a static bike, and then on the gym equipment using submaximal repetitions, at first bilateral and then unilateral. Resistance was progressively increased until the patient could not complete one repetition of the resistance applied, and the tester aimed to achieve this within seven repetitions. The final weight lifted was recorded as the patient’s 1RM. 1RM scores were later normalised to body mass (1RM/bodyweight (kg) × 100), to account for the confounding influence of body weight on strength measurements [18]. Seat position was recorded at baseline so that positioning could be replicated at weeks six and week twelve.

#### 2.7.2. EQ-5D-5L

The EQ-5D-5L survey was used to measure change of health-related quality of life. There is an extensive literature to support the validity and reliability of the EQ-5D in many conditions and populations including knee osteoarthritis [19]. The EQ-5D five-level version is a valid and reliable extension of the original three-level system [20] and may be a more useful instrument for the measurement of health status [21].

#### 2.7.3. The Patient-Specific Function Scale (PSFS)

Functional change was measured using the Patient-Specific Function Scale (PSFS), proven to be reliable, valid, and efficient in patients with knee dysfunction [22,23]. In this survey, patients identified up to five activities they had difficulty performing as a result of their knee osteoarthritis and scored their ability to complete that task before and after completion of the Kneefit programme.

#### 2.7.4. The Oxford Knee Score

The Oxford Knee Score (OKS) was used as a measure to assess knee symptoms before and after the Kneefit programme. While the OKS was originally used to assess outcomes from knee-replacement surgery, it is now widely utilised to assess patient outcomes following non-surgical interventions [24] and has demonstrated good psychometric properties [25].

### 2.8. Analysis

Data from the pre-programme and at six weeks and twelve weeks were analysed using SPSS Predictive Analytics Software version 11.5 (SPSS Inc., Chicago, IL, USA), with the significance level set to *p* < 0.05. The normality of the data was assessed using the Shapiro–Wilk test. The data from the questionnaires and 1RM maximum tests were not normally distributed, and thus the Wilcoxon Signed Rank test was used to evaluate the changes from week 1 to week 6 and week 12. Given the non-normal distribution, medians and interquartile ranges were used to describe the data [26].

## 3. Ethical Considerations

The Research Department at The Royal Bournemouth Hospital confirmed that ethical approval was not required as this study is a service evaluation. In keeping with good practice, the ethical principles for medical research outlined in the Declaration of Helsinki were followed [27].

## 4. Results

**Figure 1:**
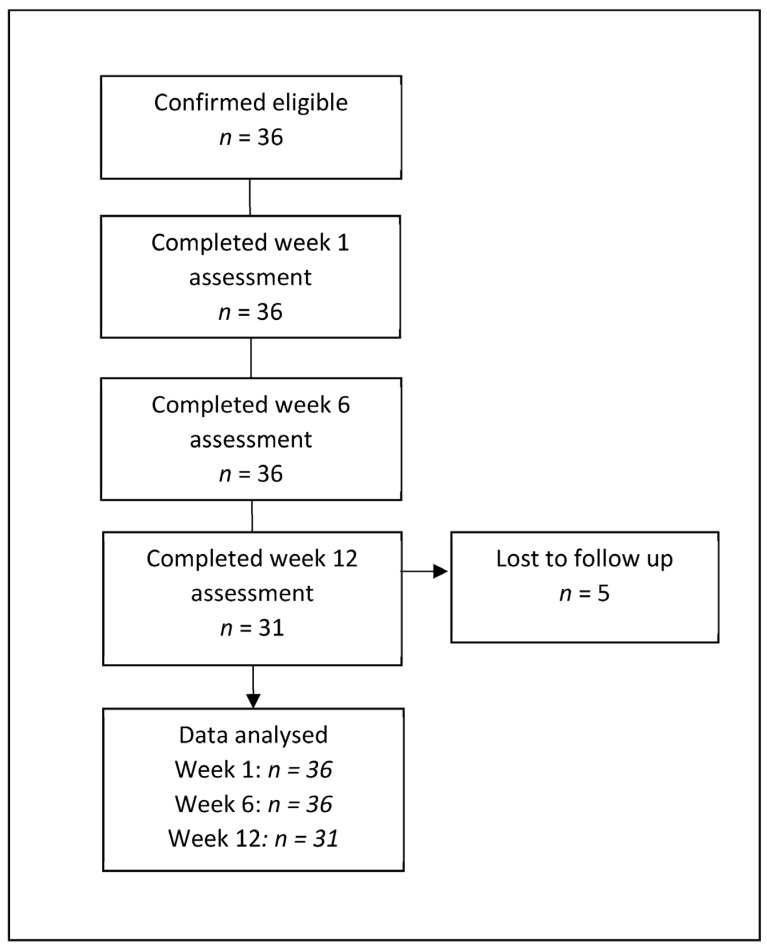
Patient recruitment and retention.

Thirty-six patients were included at baseline (week 1), and week 6, and 31 patients completed their twelve-week assessment. Five patients were lost to follow-up between weeks 6 and 12. No adverse events were recorded throughout the evaluation period and the Kneefit intervention was delivered as planned and as outlined in the methodology section. The mean age of the participants at baseline was 60 ± 7.5 years and 47% had osteoarthritis of the left knee, and 53% of the right. The mean weight of the patients was 86.8 ± 15.6 kg at baseline, which was reduced to 86.5 ± 15.6 kg at week 6, and 86.7 ± 15.0 kg at week 12. Some patients were unable to complete every outcome measure due to (i) availability of PROM (*n* = 8); (ii) availability of testing equipment (*n* = 2); and (iii) pain preventing 1RM testing on the affected limb (*n* = 5). In addition, body weight was not recorded at baseline for one patient or at week six for six patients, and therefore strength assessments could not be normalised. A flowchart of patient recruitment and retention is shown in Figure 1.

**Figure 2:**
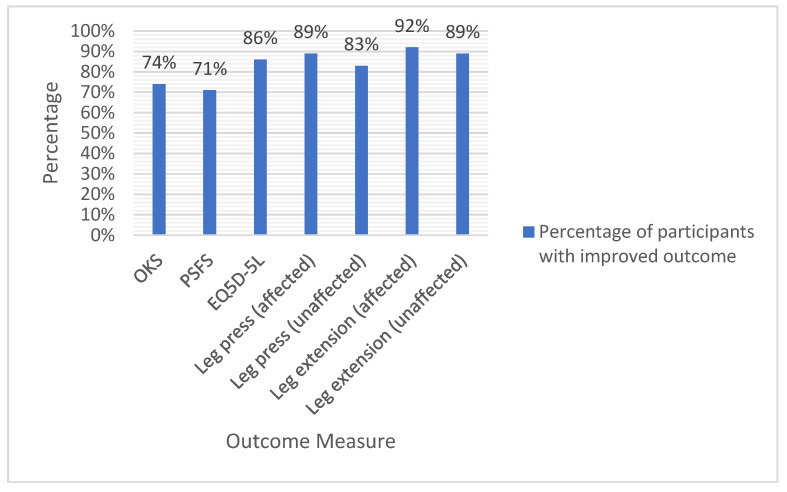
Percentage of patients with improved outcome at week 12. (OKS = Oxford Knee Score, PSFS = Patient Specific Functional Scale, EQ5D-5L = The 5-level EQ-5D version).

Table 2 demonstrates median values for the outcome measures at baseline, week 6, and week 12. Statistically significant improvements were found at 6 and 12 weeks for changes in the Oxford Knee Score (median change: 4.0, IQR 4.0 to 9.0, *p* < 0.001 and 4.0, IQR 0 to 8.0, *p* < 0.001), EQ5D-5L (median change: 0.078, IQR 0.03 to 0.20, *p* < 0.001 and 0.071, IQR 0.02 to 0.25, *p* < 0.001), and the PSFS (median change: 1.3 IQR 0 to 2.6, *p* = 0.005 and 2.3 IQR −0.3 to 3.3, *p* = 0.016), as shown in Table 3. In addition, significant improvements were found for 1RM leg-press and knee-extension scores on both the affected and unaffected legs between weeks 1 and 6, and between weeks 1 and 12 (Table 3). Figure 2 shows that at least 70% of patients had positive changes for all outcomes at week 12, with over 80% having improvements in strength.

**Figure 3:**
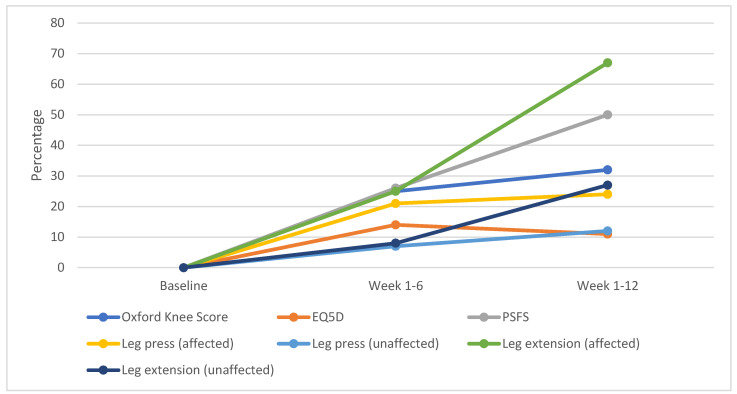
Percentage change in outcome from baseline to weeks 1 and 12 (PSFS = Patient Specific Functional Scale, EQ5D-5L = The 5-level EQ-5D version).

Figure 3 shows the percentage change in outcomes from baseline to week 1 and 12. Although patients continued to improve during weeks 6 and 12, progression slowed across the Oxford Knee Score, leg press (affected limb) and leg press (unaffected limb). EQ5D-5L scores were lower at week 12 than at week six. Changes in leg extensions (affected and unaffected limbs) and PSFS scores continued to progress at a similar rate between weeks 1 to 6 and weeks 1 to 12.

## 5. Discussion

The Kneefit programme was designed to implement global evidence-based recommendations for the conservative management of knee osteoarthritis to the local area of Bournemouth, Dorset (UK), where there is a high percentage of older adults seeking care for musculoskeletal disorders. Symptoms of knee osteoarthritis typically include joint pain accompanied by varying degrees of functional limitation and reduced quality of life. In this evaluation, we found that patients with knee osteoarthritis who took part in Kneefit demonstrated significant improvements in leg strength (1RM leg press and leg extension), osteoarthritis symptoms (Oxford knee score), function (PSFS) and quality of life (EQ5D-5L) from week one to week twelve.

The change of 0.078 (week 1 to 6) and 0.071 (week 1 to 12) in the EQ5D-5L scores meets the minimally clinically important improvement (MCII) of 0.07 previously reported for non-surgical hip and knee osteoarthritis patients [28]. Likewise, the change of 2.3 in the PSFS from weeks 1 to 12 matches the MCII of 2.3 previously reported in general populations [23]. While no data specific to osteoarthritic populations are available, the median improvement of 7.8 kg observed in leg extension 1RM from weeks 1 to 12 is likely to have clinical significance as well. A study of patients participating in community rehabilitation for chronic obstructive pulmonary disease (COPD) suggests that an improvement of 5.7 kg in 1RM is clinically relevant [29]. Data on the clinical significance of the OKS are limited to surgical populations, and therefore comparisons are not possible. Finally, we were not able to find any comparative data on the clinical significance of leg-press improvements.

## 6. Interpretation

These findings are important on a local scale and may benefit both primary and secondary healthcare systems in Dorset, where innovative care strategies are required to manage the volume of patients presenting with lower-limb musculoskeletal disorders. On a national and global scale, these findings add to the body of literature supporting strengthening exercises in people with knee osteoarthritis, and the feasibility of prescribing resistance training as a percentage of 1RM scores. In knee osteoarthritis, exercise intensity is not always specified, or prescribed in relation to 1RM testing, but is instead characterized using rate of perceived exertion (RPE) scales [30], perhaps due to concerns about pain during 1RM testing. While we found that prescribing strength exercises as a 75% 1RM to be feasible amongst the majority of this population, and effective at improving 1RM scores, five patients were unable to perform 1RM testing on their affected limbs due to pain. In addition, some patients were at first concerned about the strength-training element of the programme; however, with reassurance they bought into the programme and were motivated to achieve their prescribed exercise dose, which perhaps highlights the importance of using a personalised approach and supervision when first prescribing exercise at this intensity.

Our results are comparable to global exercise programmes designed for people with knee osteoarthritis, such as the GLA:D^®^ programme (University of Southern Denmark, Odense, Denmark) or Escape-pain, and are therefore likely to be generalisable to the wider knee osteoarthritis population. For example, patients who took part in the Escape-pain programme for knee osteoarthritis improved their mean EQ5D-5L scores from 0.73 at baseline to 0.81 after the intervention (*n* = 29) [31], similar to the change from 0.64 to 0.71 observed before and after the Kneefit programme. Likewise, data from the GLA:D^®^ programme (*n* = 28,370) found a 12–26% increase in the quality-of-life subscale of the HOOS/KOOS from baseline to immediately after treatment [32], comparable to the 32% increase in the total KOOS score observed between baseline and week twelve of the Kneefit programme.

In line with the PDSA cycle of continuous service improvement [15], this initial evaluation allowed us to identify opportunities to improve the Kneefit programme in its next iteration. While successful at delivering an effective dose of exercise, the Kneefit programme described here did not offer an educational component, as recommended by NICE, AAOS and OARSI, other than the education provided on exercising. For the next cycle of this programme, it was decided to include educational information in a standardised patient information leaflet, to include advice on other self-management strategies and pharmacological management of osteoarthritis, that could be updated as and when research evidence progresses. In addition, discussions with the delivering team found that it was difficult to fit in all the prescribed exercises during the circuit class, and therefore the class duration may need to be increased, or the number of exercises reduced, in subsequent versions of the Kneefit programme. Finally, the smaller change observed in some measures between weeks one and twelve, when compared to weeks one to six, suggest there may be room to increase the exercise dose prescribed in the home exercise programme.

## 7. Limitations

The evaluation was designed to evaluate the effects of a quality-improvement study. Nonetheless, the absence of a control group may have resulted in an overestimation of the treatment’s effect when compared to those found in controlled clinical trials. Patients were selected by physiotherapists or surgeons, and therefore outcomes for non-selected or self-referred patients may differ from the results reported here.

## 8. Conclusions

The Kneefit programme was successful at improving both functional and strength-related outcome measures in patients with knee osteoarthritis. Our findings suggest that tailoring strength exercise based on the 1RM strength-training principles is feasible in this population. Future work will involve developing the Kneefit programme to include an educational component and an advanced home-exercise programme. In addition, future evaluations will involve comparing the Kneefit programme to global models of care, such as the Escape-pain or GLA:D programme, so that its impact can be validated on a wider scale. Finally, a longer-term evaluation is required so that the long-term impact of the Kneefit programme on knee osteoarthritis symptoms can be assessed. 

## Figures and Tables

**Table 1:** The development of the Kneefit programme using the PDSA model.

PDSA Element	Description
Plan	Implement evidence-based recommendations for the self-management of knee osteoarthritis locally in Bournemouth. Improve the quality-of-care patients in Bournemouth, Dorset receive for the conservative management of knee osteoarthritis.
Do	Deliver the Kneefit programme to a pilot sample Collect data on patient-reported outcome measures (PROMS) and lower limb function.
Study	Examine the feasibility of training at 75% 1RM in patients with knee osteoarthritis. Compare patient outcomes before and after participation in the Kneefit programme.
Act	Develop the Kneefit programme to include an educational component. Develop and advance the home exercise programme. Examine patient retention and compliance with the programme.

**Table 2:** Median (IQR) of outcomes at weeks 1, 6 and 12.

Outcome Measure	*n*	Week 1 Median (IQR)	*n*	Week 6 Median (IQR)	*n*	Week 12 Median (IQR)
Oxford Knee Score	36	28.0 (24 to 35.75)	35	35.0 (26.0 to 40.0)	31	37.0 (26.0 to 42.0)
EQ5D-5L	31	0.64 (0.49 to 0.72)	31	0.73 (0.60 to 0.80)	29	0.71 (0.65 to 0.78)
PSFS	36	4.2 (2.7 to 5.5)	35	5.3 (4 to 6.6)	31	6.3 (5 to 7.6)
Leg press—affected (1RM normalised to bodyweight (kg))	34	59.3 (40.2 to 71.9)	35	71.7 (57.6 to 84.2)	29	73.3 (65.1 to 97.6)
Leg press—unaffected (1RM normalised to bodyweight (kg))	35	73.3 (54.9 to 86.8)	35	78.30 (65.6 to 98.3)	30	81.8 (75.4 to 107.0)
Leg extension—affected (1RM normalised to bodyweight (kg))	30	11.2 (3.2 to 23.6)	34	14.0 (6.5 to 29.1)	28	18.1 (10.3 to 28.7)
Leg extension—unaffected (1RM normalised to bodyweight (kg))	35	20.3 (12.1 to 32.1)	36	22.0 (11.5 to 31.1)	29	25.8 (17.7 to 25.2)

**Table 3:** Median (IQR) of changes in outcome from Week 1 to Week 12.

Outcome	*n*	Change from Week 1 to Week 6 Median (IQR)	*p*	*n*	Change from Week 1 to Week 12 Median (IQR)	*p*
Oxford Knee Score	35	4.0 (4.0 to 9.0)	<0.001	31	4.0 (4 to 8.0)	<0.001
EQ5D-5L	31	0.078 (0.03 to 0.20)	<0.001	29	0.071 (0.02 to 0.25)	<0.001
PSFS	35	1.3 (0 to 2.6)	0.005	31	2.3 (−0.3 to 3.3)	0.016
Leg press—affected (1RM normalised to bodyweight (kg))	33	17.8 (5.8 to 27.7)	<0.001	28	24.2 (9.6 to 35.9)	<0.001
Leg press—unaffected (1RM normalised to bodyweight (kg))	34	10.1 (0.6 to 19.1)	<0.001	29	13.5 (1.0 to 24.4)	<0.001
Leg extension—affected (1RM normalised to bodyweight (kg))	30	3.8 (0.1 to 9.4)	<0.001	24	7.8 (3.9 to 14.0)	<0.001
Leg extension—unaffected (1RM normalised to bodyweight (kg))	35	0.6 (−1.9 to 6.1)	0.153	38	5.4 (1.7 to 7.4)	<0.001

## Data Availability

The data are available upon request from the corresponding author.

## Supplementary Materials

The following supporting information can be downloaded at: https://www.mdpi.com/article/10.3390/jcm12093156/s1, File S1: The TIDieR (Template for Intervention Description and Replication) Checklist; File S2: CERT Checklist; File S3: The Kneefit programme; File S4: Kneefit Exercise Class Home Exercise Programme; File S5: The exercise diary.
