# Supplementary material for: A 1RM Strengthening and Exercise Programme for the Treatment of Knee Osteoarthritis: A Quality-Improvement Study
Source: J Clin Med. 2023 Apr 27;12(9):3156. doi: 10.3390/jcm12093156 (PMC10179632; doi:10.3390/jcm12093156)
Supplement: Supplementary file 1 [file jcm-12-03156-s001.zip › jcm-2301335-File S3.pdf]

NHS
The Royal Bournemouth and Christchurch Hospitals
NHS Foundation Trust

Start date: **End date:**

	Exercise		Date								
**1a**	Static bike<br>Time: 6 mins	Load:<br>Rest:									
**1b**	Rower/cross trainer<br>Time: 6 mins	Load:<br>Rest:									
**2a**	Leg press<br>3 sets, 8-12 reps	Load:<br>Rest:									
**2b**	Leg extension<br>3 sets, 8-12	Load:<br>Rest:									
**3a**	Rest<br>Time: 6 mins	Load:<br>Rest:									
**3b**	Hamstring curl (ankle weight or machine)<br>3 sets, 8-12 reps	Load:<br>Rest:									
**4a**	Sidestep (TheraBand)<br>3 sets, 8-15 reps	Load:<br>Rest:									
**4b**	Bridge/single leg bridge with mat or gym ball<br>3 sets, 10-15 reps	Load:<br>Rest:									
**5a**	Balance exercises (BOSU ball, wobble board, balance in bars)<br>Time: 6 mins	Load:<br>*Bodyweight*<br>Rest:									

Start date: **End date:**

**5b**	Calf raises (two or one leg, in bars)<br>3 sets, 10-25 reps	Load:<br>*Bodyweight*<br>Rest:									
