# Supplementary material for: A 1RM Strengthening and Exercise Programme for the Treatment of Knee Osteoarthritis: A Quality-Improvement Study
Source: J Clin Med. 2023 Apr 27;12(9):3156. doi: 10.3390/jcm12093156 (PMC10179632; doi:10.3390/jcm12093156)
Supplement: Supplementary file 1 [file jcm-12-03156-s001.zip › jcm-2301335-File S4.pdf]

The Royal Bournemouth and Christchurch Hospitals
NHS Foundation Trust

Kneefit Exercise Class Home Exercise Programme

These exercises have been designed to strengthen your legs in order to improve pain and function as part of the Kneefit group. To get the best outcome they should be performed in TWO SESSIONS per week NOT including the class.

The exercises should be performed pain free but should challenge the ability of your muscles to produce force. Perform these exercises all in one session but not on consecutive days. Do the exercises in a safe environment, using hand hold support where necessary. The exercises have been grouped into muscle group and difficulty. If you can do the first one easily, replace it with the next one in the group. Do one exercise from each group.

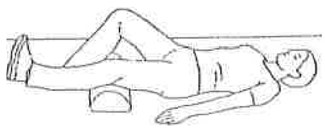

1. Quadriceps

Lying on your back or sitting. Bend one leg and put your foot on the bed and put a firm roll under the other knee.

Exercise your straight leg by pulling your foot and toe s up, tightening your thigh muscle and straightening the knee (keep knee on the roll).

Hold approx. 10 seconds and slowly relax. To make the exercise harder, put a weight around your ankle.

Repeat 10-15 times x 3 sets.

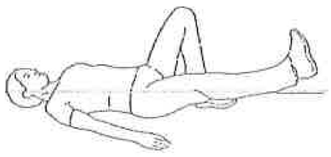

2. Quadriceps

Lying on your back with one leg straight and the other leg bent. (You can vary the exercise by having your foot pointing either upwards, inwards or outwards).

Exercise your straight leg by pulling the toes up, straightening the knee and lifting the leg 20 cm off the bed. Hold approx 5 secs - slowly relax.

Repeat 10-15 times x 3 sets

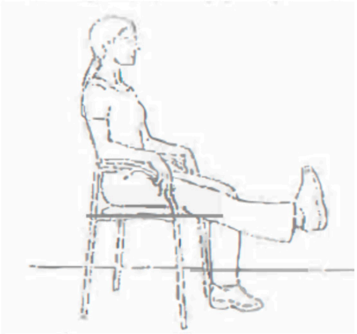

3. Quadriceps

Sit on a chair.

Pull your toes up, tighten your thigh muscle and straighten your knee. Hold for approx. 5 seconds and slowly relax your leg.

Repeat 10-15 times x 3 sets.

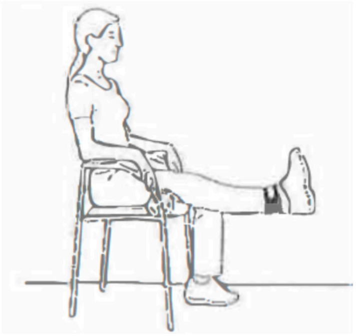

4. Quadriceps

Sit on a chair with a cushion under your knee and a ____ kg weight around your ankle.

Pull your toes up, tighten the front of your thigh muscle and straighten your knee slowly. Hope for approx. 5 seconds.

Repeat 10-15 times x 3 sets.

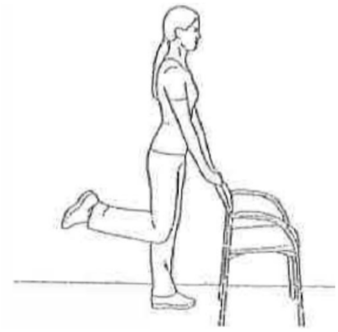

1. Hamstrings

While standing, hold onto a support and bring one leg slightly back-wards.

Bend your knee and lift your foot off the floor.

Repeat 10-15 times x 3 set.

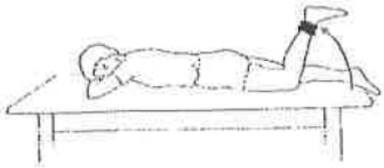

2. **Hamstrings**

Lie down as shown. Place a weight on your ankle.

Bend your knee slowly.

Hold this for 3 seconds and then slowly lower.

Repeat 10-15 times x 3 sets.

©PhysioTools Ltd

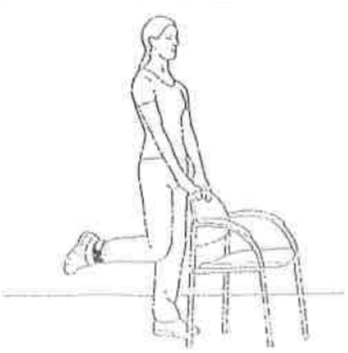

3. Hamstrings

Stand. Put a ___ kg weight around your ankle. Hold onto a support and bring one leg slightly backwards.

Bend your knee and lift your foot off the floor. Hold for ____seconds.

Repeat 10-15 times x 3 sets.

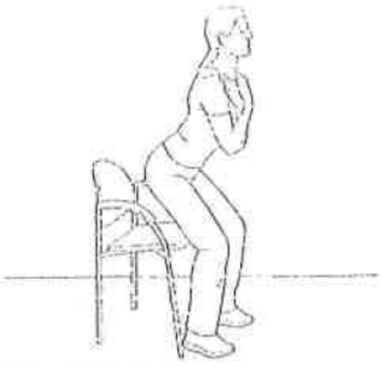

1. Whole legs

Sit with your armed crossed. Stand up and then sit down slowly on the chair.

This can be made more difficult by changing the height of the chair.

Repeat 10-15 times x 3 sets.

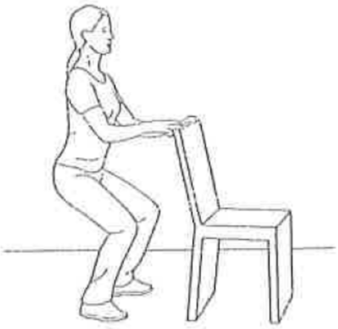

2. Whole legs

Stand behind a chair and support yourself with both hands.

Slowly bend your hips and knees, trying to push your bottom back as low as you can control. Stand up tightening your buttock muscles.

Repeat 10-15 times x 3 sets.

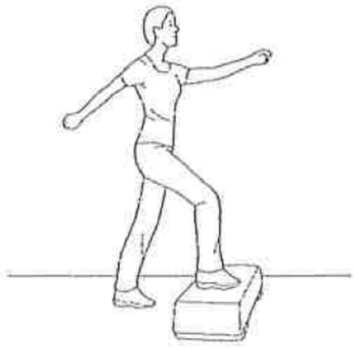

3. Whole legs

Stand in front of a 20-40 cm step

Step up ___ times with one leg leading and then repeat with the other leg leading.

Repeat ___ times.

©PhysioTools Ltd

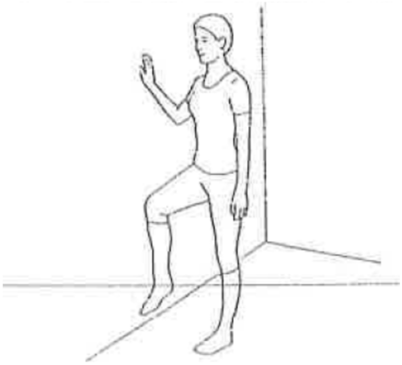

1. Balance

Stand. Lift one leg. Try to balance and hold for as long as possible using minimal hand support as needed.

Repeat 10 times.

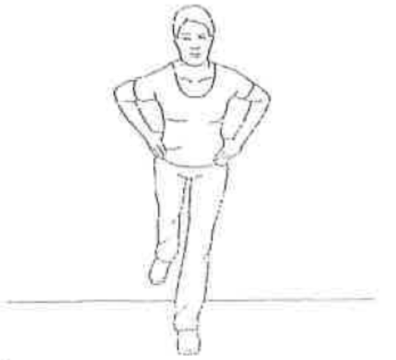

2. Balance

Stand. Slowly lean your body forward 45 degrees. At the same time, straighten one leg behind you and slightly bend the leg you are standing on. Try to hold for as long as you can.

Repeat 10 times.

1. Lateral trunk control

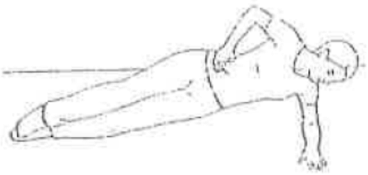

Wall plank: Stand a couple of feet away from a wall with your body and legs straight. Support your body weight on your fore-arm and feet. Try to keep your body straight.

Tighten your stomach muscles and keep your neck and back straight.

Hold 10-60 seconds. Repeat 3 times.

2. Lateral trunk control.

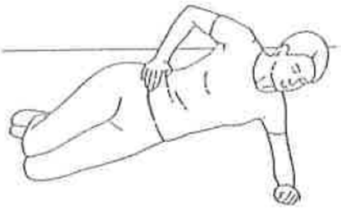

Lie on your side with your knees bent. Support your body weight on your forearm and knee.

Tighten your stomach muscles and keep your neck and back straight.

Hold for 10-60 seconds. Repeat 3 times.

©PhysioTools Ltd

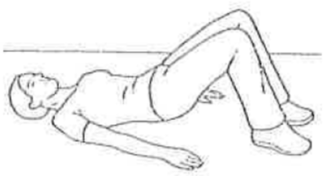

1. Bridge

Lying on your back with your knees bent.

Squeeze your buttocks together and lift your bottom off the floor. Return to starting position.

Repeat 3 x 10 -15 times.

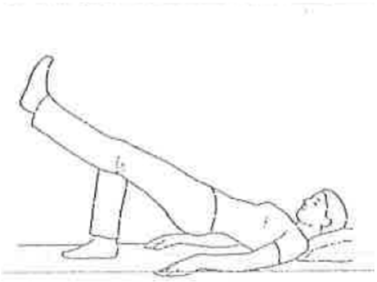

2. Bridge

Lying on your back with your knees bent.

Lift your hip up and keep it straight while shifting the weight over to one leg. Then straighten the other leg – put it back down and repeat with the other leg.

Repeat 3 x 10-15 times.

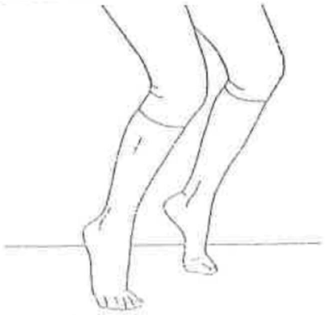

1. Calves

Sit with your feet apart.

Bend your knees and then slowly lift your heels.

Repeat 3 x 10-25. Once you can do 25, move onto the next level.

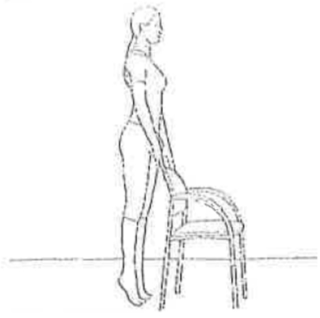

2. Calves

Stand. Push up onto your toes.

Repeat 3 x 10 – 25.

Once you can do 25 repetitions, move onto the next level.

©PhysioTools Ltd

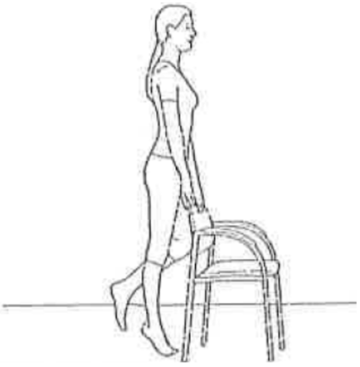

3. Calves

Stand on one leg. Push up onto your toes.

Repeat 3 x 10 – 25.

©PhysioTools Ltd
