# Supplementary material for: A 1RM Strengthening and Exercise Programme for the Treatment of Knee Osteoarthritis: A Quality-Improvement Study
Source: J Clin Med. 2023 Apr 27;12(9):3156. doi: 10.3390/jcm12093156 (PMC10179632; doi:10.3390/jcm12093156)
Supplement: Supplementary file 1 [file jcm-12-03156-s001.zip › jcm-2301335-File S5.pdf]

Week		Week 1			Week 2			Week 3			Week 4			Week 5			Week 6		
Location		Home	Home	Class	Home	Home	Class	Home	Home	Class	Home	Home	Class	Home	Home	Class	Home	Home	Class
Area	Exercise	Date																	
**Quads**	1																		
	2																		
	3																		
	4																		
**Hamstrings**	1																		
	2																		
	3																		
**Whole leg**	1																		
	2																		
	3																		
**Balance**	1																		
	2																		
**Trunk**	1																		
	2																		
**Bridging**	1																		
	2																		
**Calves**	1																		
	2																		
